# Goodpasture syndrome manifesting as nephrotic-range proteinuria with anti-glomerular basement membrane antibody seronegativity: A case report

**DOI:** 10.1097/MD.0000000000022341

**Published:** 2020-09-25

**Authors:** ZhengXia Zhong, JiaXing Tan, Yi Tang, ZhengFu Li, Wei Qin

**Affiliations:** a Division of Nephrology, Department of Medicine, Affiliated Hospital of Zunyi Medical University, Guizhou; b Division of Nephrology, Department of Medicine, West China Hospital, Sichuan University, Chengdu, Sichuan; c Department of Respiration, Affiliated Hospital of Zunyi Medical University, Guizhou, China.

**Keywords:** case report, goodpasture syndrome, negative anti-gbm antibody, nephrotic-range proteinuria

## Abstract

**Rationale:**

The Goodpasture syndrome is an extremely rare disease, with renal and pulmonary manifestations, and is mediated by anti-glomerular basement membrane (anti-GBM) antibodies. Renal pathological changes are mainly characterized by glomerular crescent formation and linear immunofluorescent staining for immunoglobulin G on the GBM. There are few reports on the atypical course of the syndrome involving serum-negative anti-GBM antibodies. Therefore, we present a case of Goodpasture syndrome that presented with nephrotic-range proteinuria and was seronegative for anti-GBM antibodies.

**Patient concerns:**

A 38-year-old Chinese man presented with a lung lesion that was discovered by physical examination a month prior to presentation. The chief concern was occasional hemoptysis without fever, cough, chest pain, and edema.

**Diagnoses:**

Laboratory testing revealed that the urinary protein level and urine erythrocyte count were 7.4 g/24 hours and 144/high-power field (HPF), respectively. Serological testing for anti-GBM antibodies was negative. Chest computed tomography revealed multiple exudative lesions in both lungs, indicating alveolar infiltration and hemorrhage. Electronic bronchoscopy and pathological examination of the alveolar lavage fluid indicated no abnormalities. However, kidney biopsy suggested cellular crescent formation and segmental necrosis of the globuli, with linear IgG and complement C3 deposition on the GBM. These findings were consistent with the diagnosis of anti-GBM antibody nephritis.

**Interventions:**

The patient underwent 7 sessions of double filtration plasmapheresis. He was also administered with intravenous methylprednisolone and cyclophosphamide. After renal function stabilization, he was discharged under an immunosuppressive regimen comprising of glucocorticoids and cyclophosphamides.

**Outcomes:**

Three months later, follow-up examination revealed that the 24-hour urine protein had increased to 13 g. Furthermore, the urine erythrocyte count was 243/HPF. After a 6-month follow-up, the patient achieved partial remission, with a proteinuria level of 3.9 g/24 hours and a urine erythrocyte count of 187/HPF.

**Lessons:**

This extremely rare case of Goodpasture syndrome manifested with seronegativity for anti-GBM antibodies and nephrotic-range proteinuria. Our findings emphasize the importance of renal biopsy for the clinical diagnosis of atypical cases. Furthermore, because renal involvement achieved only partial remission despite therapy, early detection and active treatment of the Goodpasture syndrome is necessary to improve the prognosis of patients.

## 1 Introduction

The Goodpasture syndrome is a rare autoimmune disease that is mediated by anti-glomerular basement membrane (anti-GBM) antibodies. Acute kidney failure and life-threatening pulmonary hemorrhage are typical clinical symptoms.^[1]^ Associated renal pathological changes are characterized by glomerular crescent formation on the GBM and linear immunofluorescence staining positive for immunoglobulin G. The discovery of anti-GBM antibodies in 1967 verified the pathogenesis of the Goodpasture syndrome.^[2]^ However, only few studies have investigated the atypical course of the syndrome involving serum-negative anti-GBM antibodies. We present a case of Goodpasture syndrome with serology negative for anti-GBM antibodies and manifested as nephrotic-range proteinuria. The purpose of this report is to put forward new reflections for clinicians regarding this attractive case.

## 2 Case presentation

**Figure 1:**
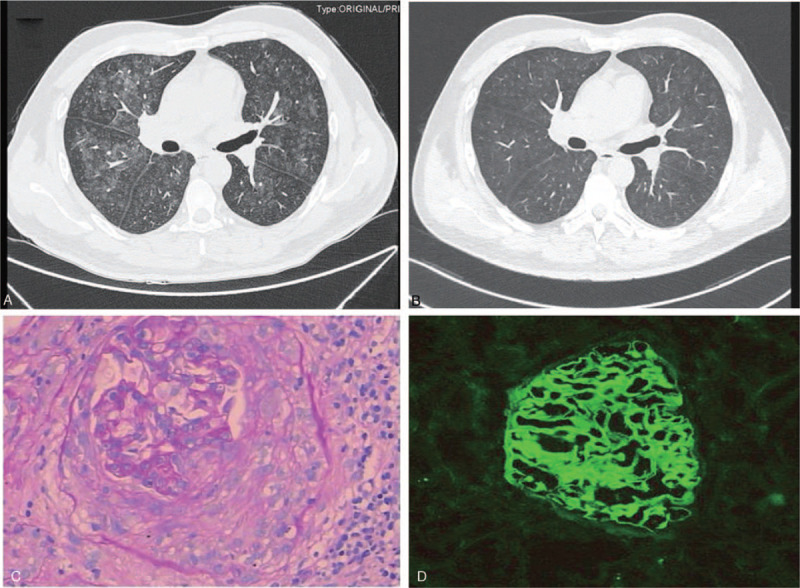
(A) High resolution CT (HRCT) indicated multiple exudation lesions of both lungs before treatment, showing alveolar infiltration and hemorrhage. (B) HRCT indicated that the pulmonary lesion got improved significantly after treatment. (C) A cellular crescent was presented in the Light microscope (PAS ×200). (D) Immunofluorescence findings showed there was linear staining along GBM with anti IgG antibody (×200).

A 38-year-old Chinese man was admitted to our hospital for a lung lesion that was discovered upon physical examination a month prior to presentation. His clinical symptoms were mild. The chief complaint included occasional hemoptysis without fever, cough, chest pain, and edema. To determine the cause, he was admitted to our medical center on July 12, 2018. The patient had a history of chronic hepatitis B. No history of hypertension, diabetes, smoking, and exposure to special drugs and poisons was reported. A physical examination of the thoracic section did not reveal any remarkable findings, except for mild edema. A chest computed tomography (CT) scan indicated multiple exudative lesions in both lungs, indicating alveolar infiltration and hemorrhage (Fig. 1A). Electronic bronchoscopy and pathological examination of the alveolar lavage fluid revealed no abnormalities. Laboratory tests revealed that the hemoglobin level, serum creatinine level, estimated glomerular filtration rate (eGFR), serum albumin level, urinary protein level, and urine erythrocyte count were 104 g/L, 71 μmol/L, 113.0 ml/minute/1.73 mm^2^, 40.7 g/L, 7.4 g/24 hours, and 144/HPF (shown Table 1), respectively. Tests for hepatitis B virus (HBV) surface antigen and deoxyribonucleic acid (HBV DNA) were positive. Immunological tests for antinuclear antibodies, anti-double stranded DNA antibodies, anti-GBM antibodies, and anti-neutrophil cytoplasmic antibodies (ANCA) were negative. The serum complement levels (C3 and C4) were normal. Ultrasonographic examination of the kidneys revealed an enhanced echo of the parenchyma in both kidneys. Renal biopsy indicated cellular crescent formation and segmental necrosis of the globuli with linear IgG and complement C3 deposition on the GBM (Fig. 1C and D). Electron microscopy indicated no electron-dense deposits. Therefore, he was diagnosed with the Goodpasture syndrome with crescentic glomerulonephritis and alveolar hemorrhage.

**Table 1:**
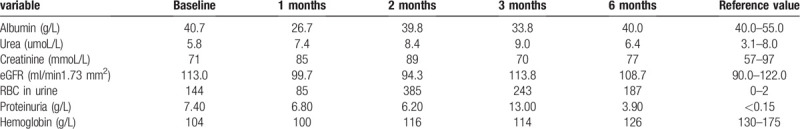
The laboratory findings of the patient.

Based on the renal pathology and laboratory findings, double filtration plasmapheresis (DFPP; once daily for 7 successive days), pulse methylprednisolone therapy (10 mg/kg daily for 3 consecutive days), and pulse cyclophosphamide treatment (1 g) were initiated. Simultaneously, entecavir was administrated to reduce HBV replication. Following DFPP, chest CT was repeated, which revealed that the pulmonary lesion had almost disappeared (Fig. 1B). Considering the stabilized renal function, he was discharged from the hospital under an immunosuppressive regimen including glucocorticoids (prednisone, 50 mg daily) and cyclophosphamide (50 mg, twice daily). The patient was followed up at the out-patient department every month. Three months later, the 24-hour urine protein level was observed to have increased to 13 g. Furthermore, the urine erythrocyte count, serum albumin, serum creatinine level, and eGFR were found to be 243/HPF, 33.8 g/L, 70 μmol/L, and 113.8 ml/minute/1.73 mm^2^, respectively. Tests for anti-GBM antibodies, ANCA, and HBV-DNA remained negative. Thereafter, prednisone was tapered down to 30 mg daily. Cyclophosphamide was replaced with tacrolimus (1.5 mg, twice daily). At the last follow-up (6th month), the serum albumin, serum creatinine level, proteinuria level, urine erythrocyte count, and eGFR were 40 g/L, 77 μmol/L, 3.9 g/d, 187/HPF, and 108.7 ml/minute/1.73 mm^2^, respectively. The patient provided informed consent for the inclusion of his data in the present report.

## 3 Discussion

The Goodpasture syndrome is an autoimmune disorder mediated by serum anti-GBM antibodies and is characterized by rapidly progressive crescent glomerulonephritis and pulmonary hemorrhage.^[3]^ It was first reported by Pasture in 1919.^[4]^

The disease is caused by anti-GBM antibodies targeted against the non-collagenous domain (NC1) of the alpha 3 chain (a3) of type IV collagen in the lungs and kidneys.^[5]^ The a3(IV) chain is mainly expressed in a few specialized basement membranes like the GBM. The anti-GBM antibody binds to the a3(IV) NC1 domain and deposits in the basement membranes of the kidneys and the lungs, subsequently activating the complement system that can result in tissue damage. Although the pathogenesis of the disease in the kidneys and the lungs is the same, the degree of damage is markedly different between them. It was reported that two-thirds of the patients with anti-GBM antibodies could progress to the Goodpasture syndrome, and one-third present with anti-GBM antibody nephritis without pulmonary manifestations.^[6,7]^ Clinically, enzyme-linked immunosorbent assay (ELISA) is a common method for the detection of anti-GBM antibodies, especially if a kidney biopsy is not performed, and has high sensitivity (>95%) and specificity (>97%). However, as previously reported, certain cases of the Goodpasture syndrome are seronegative for anti-GBM antibodies, and the reasons for the same have not been elucidated.^[8]^

In our case, the patient was seronegative for ANCA and anti-GBM antibodies, and presented with the Goodpasture syndrome with nephrotic-range proteinuria and normal renal function. While this condition may clinically be misdiagnosed as membranous nephropathy and membranous proliferative glomerulonephritis, among others, renal pathology revealed crescent formation and necrotic changes, thereby enabling a confirmative diagnosis. Immunofluorescence staining also indicated a linear deposition of immunoglobulin G and complement C3 on the GBM. Considering that the impairment of the lungs was due to alveolar hemorrhage, the patient was diagnosed with atypical Goodpasture syndrome. There are 2 relatively rare aspects of our case. Usually, classical anti-GBM disease presents with rapidly progressive kidney failure;^[9]^ however, one of the key characteristics of our report is the nephrotic-range proteinuria without renal dysfunction, which indicates a more severe degree of podocyte injury as compared to in the classic form. Nasr et al^[10]^ recently reported 7 patients who presented with nephrotic syndromes presumed to be atypical GBM diseases; however, the presence or absence of serum anti-GBM antibodies was not described in these cases. Liang et al^[8]^ also reviewed the clinical pathological features of antibody-negative anti-GBM disease, and observed that 7 out of 19 patients presented with nephrotic-range proteinuria; however, it is unclear whether the kidney function was impaired. Therefore, we believe that our report, which details a case seronegative for anti-GBM antibodies and presenting with nephrotic-range proteinuria without kidney failure, is fairly rare.

The mechanism behind undetectable circulating anti-GBM antibodies remains unclear; although there may be several explanations to this. Firstly, it is possible that the result was falsely negative due to the limitations of the test. Even though ELISA has a positive predictive value of 95%, antibody detection remains difficult in a small number of patients. It was reported the auto-antibody antigens included the α3NC1–α5NC1 domains and the linear type IV collagen epitopes^[11]^; this demonstrates that renal tissues may have different epitopes than those present in the native kidney. Secondly, the seronegativity for anti-GBM antibodies may be attributed to their shorter half-life in circulation than in the renal tissue; the antibodies may have disappeared from the circulation while collecting the blood during testing.^[12]^ Finally, antibodies with high affinity are removed from the patient's plasma by binding to the kidney and the alveolar basement membranes. Conversely, the circulating serum antibodies may have a lower affinity than those in the renal and lung tissues; therefore, these may have washed off more easily during the experimental rinsing step. Hence, it is rather important to perform a renal biopsy in highly suspicious patients that are seronegative for anti-GBM antibodies, even though the clinical manifestations are atypical.

The classical treatment regimens for the Goodpasture syndrome include plasma exchange, corticosteroids, and immunosuppressive therapy. Plasma exchange could eliminate the circulating antibodies. Corticosteroids and immunosuppressants could prevent the formation of anti-GBM antibodies. In our case, the patient received plasma exchange therapy, intravenous methylprednisolone (followed by oral prednisolone), and cyclophosphamide. These treatments relieved the lung lesion remarkably and ceased hemoptysis. However, only partial remission was achieved in case of renal involvement. The renal function was preserved and the proteinuria level was decreased by more than 50%. Considering that the Goodpasture syndrome could also lead to podocytopathy, tacrolimus was chosen for maintenance treatment. Our observations indicate that Goodpasture syndrome with nephrotic-range proteinuria may require a longer therapy and follow-up to determine prognosis.

In conclusion, we have reported the case of a patient who was diagnosed with antibody-negative Goodpasture syndrome accompanied by nephrotic-range proteinuria and normal kidney function. This presentation is different from that of classical cases. This report serves as a reminder for clinicians to maintain a high degree of awareness for identifying such uncommon cases.

## Author contributions

**Conceptualization:** Wei Qin.

**Data curation:** ZhengXia Zhong, JiaXing Tan.

**Investigation:** ZhengXia Zhong.

**Project administration:** ZhengFu Li.

**Writing – original draft:** ZhengXia Zhong.

**Writing – review & editing:** Yi Tang Wei Qin.
